# Physical virology: From virus self‐assembly to particle mechanics

**DOI:** 10.1002/wnan.1613

**Published:** 2020-01-20

**Authors:** Pedro Buzón, Sourav Maity, Wouter H. Roos

**Affiliations:** ^1^ Moleculaire Biofysica Zernike Instituut, Rijksuniversiteit Groningen Groningen The Netherlands

**Keywords:** mechanical properties, molecular virology, particle mechanics, physical virology, virus self‐assembly

## Abstract

Viruses are highly ordered supramolecular complexes that have evolved to propagate by hijacking the host cell's machinery. Although viruses are very diverse, spreading through cells of all kingdoms of life, they share common functions and properties. Next to the general interest in virology, fundamental viral mechanisms are of growing importance in other disciplines such as biomedicine and (bio)nanotechnology. However, in order to optimally make use of viruses and virus‐like particles, for instance as vehicle for targeted drug delivery or as building blocks in electronics, it is essential to understand their basic chemical and physical properties and characteristics. In this context, the number of studies addressing the mechanisms governing viral properties and processes has recently grown drastically. This review summarizes a specific part of these scientific achievements, particularly addressing physical virology approaches aimed to understand the self‐assembly of viruses and the mechanical properties of viral particles. Using a physicochemical perspective, we have focused on fundamental studies providing an overview of the molecular basis governing these key aspects of viral systems.

This article is categorized under:

Biology‐Inspired Nanomaterials > Protein and Virus‐Based Structures
Nanotechnology Approaches to Biology > Nanoscale Systems in Biology

## Graphical abstract

Viruses are highly ordered supramolecular complexes that have evolved to propagate between related organisms. Although viruses are very diverse, they share common functions and properties. In this review, we aim to provide an overview of virus self‐assembly and viral particle mechanics, summarizing the latest scientific achievements within these areas.

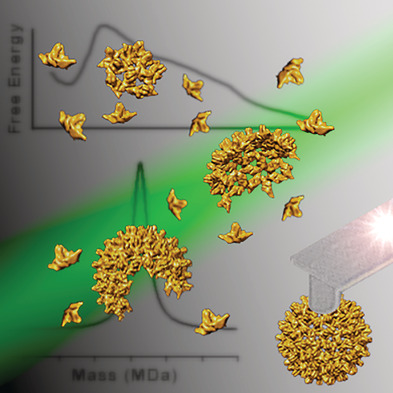

## 1 INTRODUCTION

Viruses infect cells in all kingdoms of life and, from a physicochemical perspective, can be regarded as molecular machines that have successfully evolved to spread between related organisms. They hijack their host cell's machineries in a highly efficient and minimalistic manner, in order to ensure their propagation. The molecular mechanisms behind the viral life cycle are not only complex, these processes also require a remarkably low number of essential viral components to be successful. The genetic information is stored in all possible configurations known in biology: positive‐ or negative‐sense single‐stranded (ss), or double‐stranded (ds) RNA or DNA genomes. Viruses are found with very diverse morphologies, but helical and spherical (usually icosahedral symmetry) are the most commonly found morphologies (Crick & Watson, 1956; Zlotnick, 2004).

A characteristic that many viruses have in common, is the ability to form hollow protein shells (capsids), protecting their genome from the external environment. These capsids are either formed without a genome and after completion of the shell the genome is encapsidated with the help of an ATP driven packaging motor, or the capsids are directly formed around the genome (Cuervo, Dauden, & Carrascosa, 2013). In either way, viruses have the remarkable capacity to perform this capsid formation process without any external energy source or specific assistance from the host cell. Under the right conditions, the shell is formed spontaneously by the capsid proteins (CPs), solely due to CP–CP and, in the case of assembly around the genome, CP–genome interactions. Hereby a highly organized supramolecular structure is formed. This process is known as viral self‐assembly, and it was first reproduced in vitro for a rod‐like helical virus, Tobacco mosaic virus (TMV), from its purified genome and CP (Fraenkel‐Conrat & Williams, 1955). Twelve years later, the first in vitro assembly of an icosahedral virus, Cowpea chlorotic mottle virus (CCMV), was reported (Bancroft & Hiebert, 1967). These early experiments showed that complex viral processes could be studied under controlled nonnative conditions, opening new possibilities to investigate their molecular mechanisms from a physicochemical perspective.

Besides their efficient assembly mechanism, viruses also present unique mechanical properties. During the different stages of infection, the viral capsid undergoes changes switching from highly stable states, protecting the genome, to unstable states facilitating genome release. Thus, capsids play a major role in the viral life cycle, and an understanding of their meta‐stability and conformational plasticity is key to deciphering the mechanisms governing the successive steps in viral infection. In addition, viral mechanical properties, such as elasticity/deformability, brittleness/hardness, material fatigue, and resistance to osmotic stress, are of particular interest in many areas beyond virology; for instance, soft matter physics, (bio)nanotechnology, and nanomedicine (Cordova, Deserno, Gelbart, & Ben‐Shaul, 2003; de Pablo, 2018; Jimenez‐Zaragoza et al., 2018; Roos, Bruinsma, & Wuite, 2010).

Both the studies on virus assembly as well as on mechanics have provided essential insights supporting the development of new approaches in nanobiotechnology and nanomedicine (Steele et al., 2017), such as the design of novel broad‐spectrum antiviral strategies (Nair et al., 2018; Vahey & Fletcher, 2019; Yang & Lu, 2018), viral‐based drug delivery systems (Lam & Steinmetz, 2018), and de novo design of synthetic protein cages with potential biotechnological or therapeutic applications (Butterfield et al., 2017; Hernandez‐Garcia et al., 2014; King et al., 2012). In this review, we aim to provide an overview of virus self‐assembly and viral mechanics, summarizing the latest scientific achievements within these areas. In the first part, we focus on recent studies on the characterization of assembly intermediates and their associated pathways, and proposed strategies to understand genome specificity during packaging. Next, we address recent results obtained from mechanical studies on viral particles, correlating mechanical properties with the different stages of the viral life cycle.

## 2 VIRUS SELF‐ASSEMBLY

In the simplest scenario of capsid formation, the functional capacity to self‐assemble resides in the primary amino acid sequence of the CPs and, hence, the folded structure of the viral protein subunits. Thus, the assembly process is solely driven by protein–protein and, for co‐assembly with viral nucleic acids, protein–genome interactions. In 1959, J. D. Bernal wrote in relation to structural units in abiogenesis: “The probability of formation of a highly complex structure from its elements is increased, or the number of possible ways of doing it diminished, if the structure in question can be broken down in a finite series of successively smaller substrates” (Bernal, 1959). This is a statement that can well be applied to virus formation, as viral capsids usually assemble from many, often hundreds, of identical proteins; as a common strategy to achieve the ultimate goal of producing new virions. One of the main challenges of this process is that all viral proteins must encounter and assemble in the crowded environment of cells, where ~200 mg/mL of irrelevant, cellular, proteins are present (Milo, 2013). An additional challenge to capsid formation is the fact that the packaging must be selective to encapsidate the viral genome, discriminating between cellular and viral genetic material, thus ensuring infectivity. Clearly, viruses have found strategies to overcome these challenges, and recent literature has reviewed different aspect of viral assembly (Bhella, 2018; Comas‐Garcia, 2019; Fernandez de Castro, Tenorio, & Risco, 2016; Garmann, Comas‐Garcia, Knobler, & Gelbart, 2016; Mateu, 2013; Perlmutter & Hagan, 2015a; Twarock & Stockley, 2019; Wang, Mukhopadhyay, & Zlotnick, 2018). The aim of this section is to review the latest scientific achievements in understanding the molecular mechanisms underlying the self‐assembly of viruses. Particularly, we focus on results of the characterization of new assembly intermediates and their associated pathways, and the latest discoveries in the viral mechanisms that provide genome specificity during packaging.

### 2.1 Pure capsid protein assembly pathways and their intermediates

**Figure 1:**
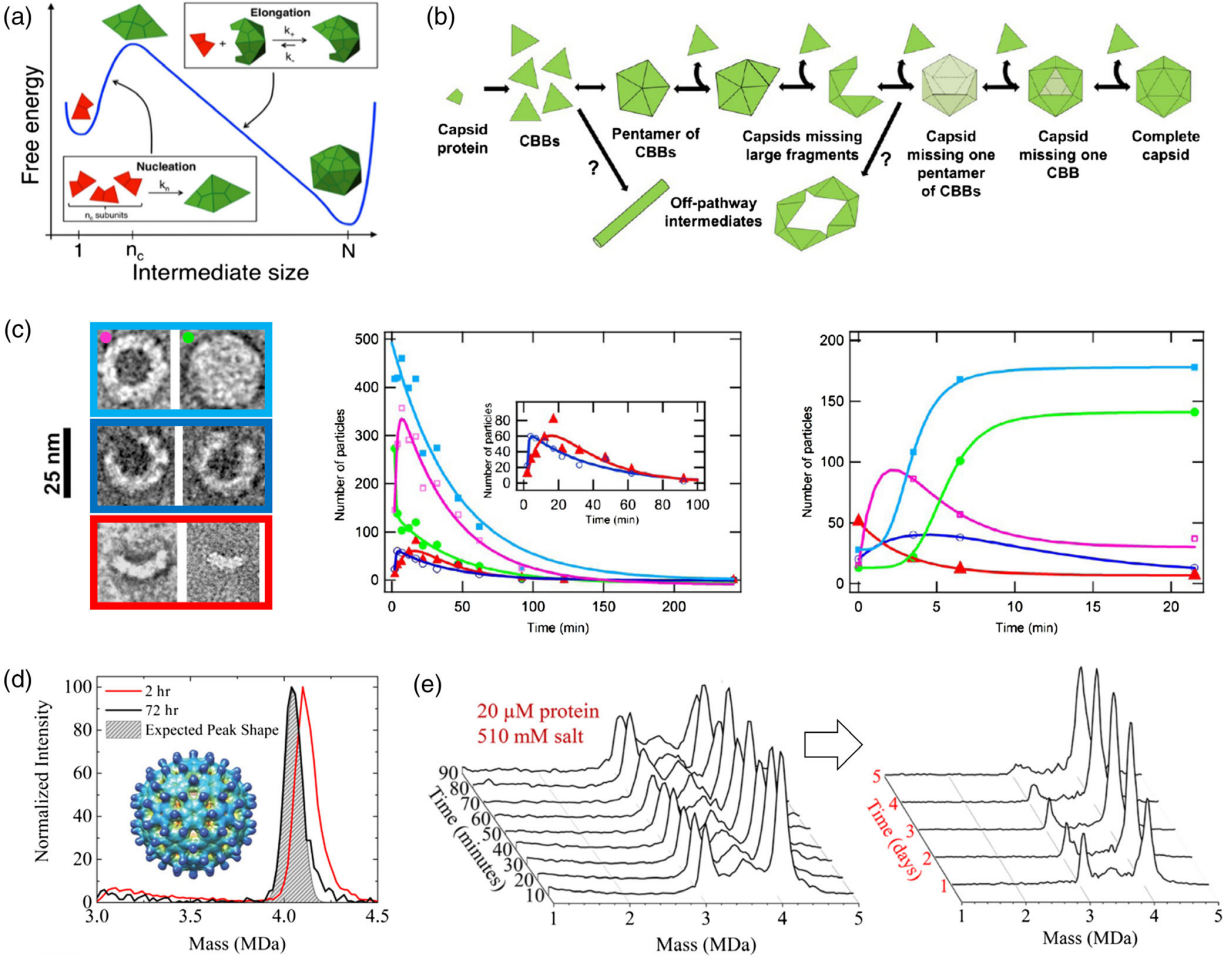
Assembly of empty particles through the nucleation, growth, and completion pathway. (a) Schematic representation of the free energy profile of the nucleation‐and‐growth/elongation pathway: first, nuclei are formed; then, the reaction proceeds downhill until the complete closure of the capsid. (Reprinted with permission from Michaels, Bellaiche, Hagan, and Knowles (2017)). (b) Self‐assembly model proposed for MVM empty capsids based on the sequential addition of trimeric subunits, or CBBs (capsid building blocks). (Reprinted with permission from Medrano et al. (2016)). (c) MVM particles imaged by TEM (left): light blue, Types I + II particles (complete capsids); green, Type I (complete capsids in basal state); magenta, Type II (complete rearranged capsids); blue, Type IIIA (large incomplete capsids); red, Type IIIB (smaller incomplete capsids). Progression of the total number of particles during disassembly (left graph) and assembly (right graph) over time. (Reprinted with permission from Medrano et al. (2016)). (d) CDMS spectrum in the region of 3.0 to 4.5 MDa after 2 hours (red trace) and 72 hours (black trace) from the initiation of the HBV assembly reaction with 5 μM CP dimers in 210 mM ammonium acetate. The gray shaded area shows the expected peak for the T = 4 capsids. Inset, representation of the HBV T = 4 capsid. Reprinted with permission from (Lutomski et al., 2017). (e) Time‐resolved CDMS spectra showing the progression of capsid assembly over the first 90 min (left) and in the scale of days (right), for an assembly reaction containing an initial CP dimer concentration of 20 μM in 510 mM ammonium acetate. (Reprinted with permission from Lutomski et al. (2018)). CBB, capsid building block; CDMS, charge detection mass spectrometry; CP, capsid protein; MVM, minute virus of mice; TEM, transmission electron microscopy

The self‐assembly of a set of viral proteins into a regularly shaped capsid involves a fine‐tuned range of interactions between the capsid proteins to create this highly ordered, symmetrical, supramolecular structure. The mechanism of assembly of viral capsids was initially characterized for the procapsid of P22 by two steps: nucleation‐and‐growth/elongation (P. E. Prevelige Jr., Thomas, & King, 1993). First, a nucleus must be formed. A nucleus is defined as the smallest intermediate of assembly with more than 50% probability to grow instead of disassembling (Perlmutter & Hagan, 2015a). Next, the nucleus size is increased by the sequential addition of protein subunits, that is, the growth phase (Figure 1a,b). In the particular case of spherical viruses, the end of the growth phase is completion or closure of the capsid. As this closure shows significant differences in kinetics and stability compared to the growth phase, it can be considered as a separate step. Thus, a three‐step mechanism was proposed and this distinction is now often used, that is: nucleation, growth, and completion (Endres & Zlotnick, 2002; Hagan, 2014; Hagan & Elrad, 2010; Michaels et al., 2017; Nguyen, Reddy, & Brooks 3rd., 2007; Zandi, van der Schoot, Reguera, Kegel, & Reiss, 2006).

In a simplified view, assembly is a spontaneous process driven by weak protein–protein interactions on the order of several *k*_*B*_ *T* (Ceres & Zlotnick, 2002). These weak attractive interactions are mainly of a hydrophobic nature (del Alamo & Mateu, 2005; Kegel & van der Schoot, 2006) and are able to overcome the entropic penalty associated with forming the highly organized capsid structures. Coarse‐grained models indicate that the growth phase proceeds as a downhill process, while completion is rate‐limited due to steric effects (Hagan, 2014; Michaels et al., 2017; Nguyen et al., 2007; Figure 1a). The latter is even thermodynamically unfavorable when only looking at the loss in flexibility upon capsid closure (Nguyen et al., 2007). Under assembly conditions, small intermediates are transient and the formation of nuclei are rare events (Endres & Zlotnick, 2002; Hagan & Elrad, 2010), making them difficult to characterize. On the other hand, large intermediates are reasonably stable, and easier to identify. However, the large number of protein subunits per capsid (for icosahedral capsids typically specific multitudes of 60, as described by the quasi‐equivalence theory and the related triangulation number [Caspar & Klug, 1962]) leads to limitations in the experimental resolution. For such large numbers of subunits, it is hard to discriminate complete capsids from capsids missing a few subunits. Indeed, both nucleation and completion are very challenging processes to investigate, which explains why only a limited amount of experimental studies have been reported on this topic.

Another characteristic of virus self‐assembly studies is the fact that assembly/disassembly reactions are typically triggered under controlled conditions. The capability of CPs to assembly is frequently tuned by changing solvent conditions such as pH, salt concentration, or mild concentrations of denaturant agents. Typically, denaturant agents such as urea and guanidinium hydrochloride are used to trigger capsid disassembly, while keeping CPs in their folded state. Changing pH can also be effective at producing individual protein subunits from viral capsids. Then, assembly reactions are usually initiated by removing the denaturant agents and tuning salt concentration and/or pH to allow CPs to reassemble into capsids.

The in vitro assembly and disassembly of one of the simplest virus particles known, the minute virus of mice (MVM), has been recently characterized using a combination of atomic force microscopy (AFM) and transmission electron microscopy (TEM; Medrano et al., 2016). MVM forms *T* = 1 icosahedral capsids of 25 nm made of only 60 CPs, which arrange as trimeric subunits (Figure 1b). The whole range of particle sizes were found in this study (Medrano et al., 2016), from complete capsids, via large incomplete capsids, to small capsid intermediates (Figure 1c). Complete capsids were analyzed separately according to their capability to be permeable to uranyl ions, as characterized by TEM experiments. Figure 1c shows the disassembly (left graph) and assembly (right graph) evolution of intermediates to and from complete capsids over time. In all cases, both large and small intermediates are only populated in low numbers, and hence, transient. This is indicative of a highly cooperative process. MVM assembly represents a distinct experimental example of the nucleation‐and‐growth pathway introduced above; where capsid formation can be simplified as the sum of equilibria by which individual protein subunits are sequentially added to the growing structure until completion (Figure 1b). For MVM, and also in the following set of descriptions, we are considering the formation of empty capsids, that is, without accounting for the contribution of the genome to the assembly process. The study of such simplified systems, empty virus‐like particles (VLPs), has been proven to be instrumental in characterizing viral assembly. This is particularly relevant for viruses such as hepatitis B virus (HBV), which produces in vivo around 90% of the viral particles as empty particles (Sakamoto et al., 1983). In this context, Uetrecht and coworkers used native Mass Spectrometry (native MS) to study the nucleation mechanism of empty HBV particles (Uetrecht, Barbu, Shoemaker, van Duijn, & Heck, 2011). HBV forms *T* = 4 icosahedral particles, of 120 CP dimers, as the major product of assembly (Crowther et al., 1994; Stannard & Hodgkiss, 1979), with an expected mass of ~4 MDa (Uetrecht et al., 2008).

Using a different MS approach, the mechanisms behind the completion phase of HBV assembly was studied (Lutomski et al., 2017) by applying charge detection mass spectrometry (CDMS). Using single‐particle CDMS, the authors identified a novel mechanism by which HBV particles seem to experience overgrowth, and then slowly relax to the final *T* = 4 structure (Lutomski et al., 2017; Figure 1d). These results suggest an unexpected completion pathway for HBV, in which late intermediates are bigger in size than the complete and closed capsid. Instead of particles missing CP subunits, HBV forms slightly overgrown particles of >4 MDa (>120 CP dimers) that seem to be kinetically favorable. Then, a spontaneous proofreading process, which occurs on a time scale much longer than that of the initial assembly reaction, appears to correct overgrown structures to form icosahedral capsids (Lutomski et al., 2017). In a later study, additional CDMS experiments were performed to track HBV assembly at different ionic strength conditions (Lutomski et al., 2018). At high salt concentration (510 mM ammonium acetate) the assembly proceeds relatively fast (less than a minute), due to screening of the electrostatic repulsive interactions between CPs. However, in this situation approximately half of the assembly reaction ends in intermediates of <120 CP dimers (between 3 and 4 MDa; Figure 1e). Potentially, these are kinetically trapped intermediates formed by defective growing capsids that arise from the stronger CP–CP association energy imposed by the experimental conditions. This is in agreement with the expectations of the strengthening of CP–CP interactions by increasing salt concentration, as these interactions are modulated by competing actions of hydrophobic attractive patches, and electrostatic repulsive amino acid residues (del Alamo & Mateu, 2005; Kegel & van der Schoot, 2006). Interestingly, some of these large intermediates are part of a successful assembly pathway, since some of them evolve to full capsids, again, on a much longer time scale (Lutomski et al., 2018; Figure 1e).

Similar results have been found using single‐particle resistive‐pulse sensing (Zhou et al., 2018), at even higher salt concentration of 1.0 M. The authors identified a shift in size for late intermediates, from 105–113 CP dimers to 114–117 CP dimers, after a two‐day reaction; consistent with mass spectrometry data (Lutomski et al., 2018). In addition to these findings, it has been observed that for HBV disassembly experiments a spherical structure with a single hole has a low probability to be formed (Lee et al., 2017). However, the results presented do not provide an explanation for the observation that HBV intermediates are stalled between 3 and 4 MDa (90–120 CP dimers) under increasing ionic strength, while up to 3 MDa the reaction occurs fast. Nevertheless, these experiments shed light on the fact that a multitude of pathways are allowed during viral assembly; pathways that are partially determined by external conditions. Moreover, new insights into the complexity of the completion phase of spherical viruses are provided.

### 2.2 Effect of the RNA genome on assembly pathways

**Figure 2:**
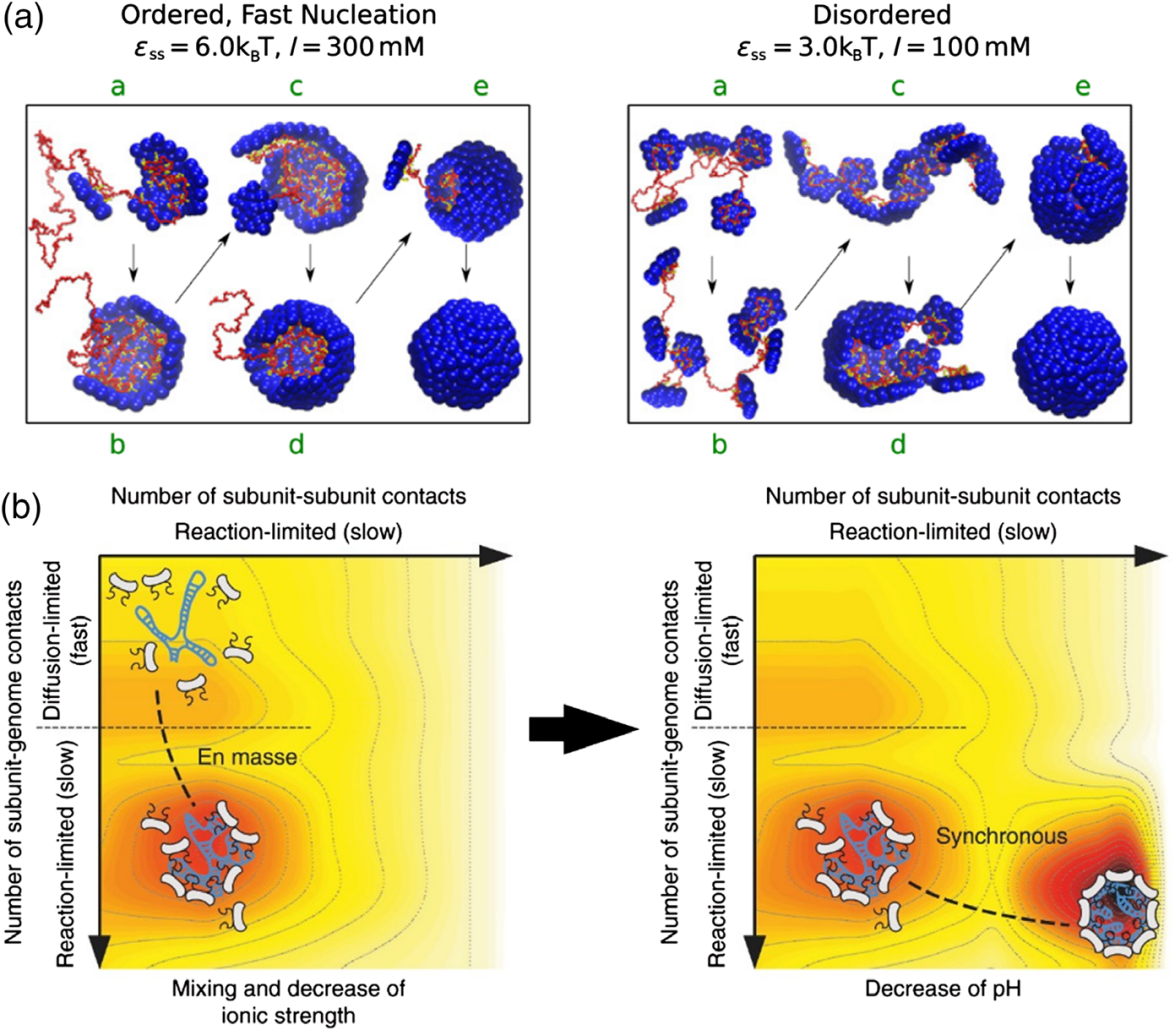
Energetics of the self‐assembly of genome‐filled capsids. (a) Snapshots obtained from simulations of the nucleation‐and‐growth (ordered) and en masse (disordered) assembly pathways by tuning the parameters ε_SS_ (protein–protein interaction strength) and I (ionic strength). (Reprinted with permission from Perlmutter et al. (2014)). (b) Free energy landscape scheme of CCMV assembly modulated by the change in ionic strength (left) and pH (right). Left, the graph shows the formation of a CP‐genome amorphous complex through the en masse pathway, driven by CP–genome interactions. Right, the amorphous complex rearranges into a full capsid by the increased strength of CP–CP interactions through the synchronous pathway. Dark colors delimit areas of low free energy, while light colors represent high free energies regions. (Reprinted with permission from Chevreuil et al. (2018)). CCMV, Cowpea chlorotic mottle virus; CP, capsid protein

We have seen how the assembly pathway of empty VLPs can be modulated by tuning the association energy between CP subunits. We now consider the role of the genome, which can redefine the viral assembly pathway. Specially, we focus on the mechanisms that lead to the assembly of small spherical viruses around (ss) genomes. The viral assembly around single‐stranded genomes is widely accepted to be thermodynamically driven by electrostatic interactions between a negatively charged genome and positively charged CPs (H. K. Lin, van der Schoot, & Zandi, 2012; Sikkema et al., 2007; Sivanandam et al., 2016; Zlotnick, Aldrich, Johnson, Ceres, & Young, 2000). For instance, CPs of many negative‐sense ssRNA viruses interact with RNA via a positively charged cleft (Ruigrok, Crepin, & Kolakofsky, 2011), while for a variety of positive‐sense RNA viruses, CPs present a flexible arginine‐rich motifs (Speir, Munshi, Wang, Baker, & Johnson, 1995). Taking this into account, it is now clear that the relative balance between CP–CP and CP–genome interaction energies, will determine the assembly path. Focusing on these parameters, two different pathways have been proposed (Elrad & Hagan, 2010; Perlmutter, Perkett, & Hagan, 2014; Zlotnick, Porterfield, & Wang, 2013): (a) in the first one, a nucleation structure with defined CP–CP interactions is formed on the genome, and the nucleus grows by the sequential addition of subunits; (b) in the second one, subunits absorb fast and en masse onto the genome with loose or absent CP–CP interactions, and then the irregular complex reorganizes into a well‐ordered, filled capsid. The former resembles the nucleation‐and‐growth mechanism described for empty capsids, and is associated with strong CP–CP interactions. The latter pathway instead, is preferentially followed by viruses with stronger CP–genome interactions (Figure 2a).

Cowpea chlorotic mottle virus is an icosahedral, ssRNA, plant virus that forms *T* = 3 capsids by the association of 90 CP dimers. CCMV in vitro assembly has been characterized using a two‐step assembly reaction, as recently reviewed (Garmann et al., 2016). It assembles by the en masse pathway and the two‐step assembly reaction proceeds as follows: first, CPs bind to the genome forming amorphous particles at low ionic strength and neutral pH; then, the pH is lowered to enhance CP–CP contacts and regular *T* = 3 filled VLPs are formed. Chevreuil and coworkers followed this assembly on ssRNA using time‐resolved small angle X‐ray scattering (Chevreuil et al., 2018). At neutral pH, the authors identified stable intermediates of around 75 CP subunits in size, less than the expected 90 dimers that would form a complete capsid. Following the number of genome‐bound subunits per intermediate over time, a single exponential growth function revealed a genome binding time *τ*_bind_ of ~28 ms. Analogously, tracking the radius of gyration over time, indicative of the compactness of the formed structures, the time scale of forming the final compact structure *τ*_struc_ was determined to be ~48 s; three orders of magnitude higher than *τ*_bind_. This points toward an en masse pathway, in agreement with the expected CCMV assembly mechanism (Figure 2a, right panel). However, the authors report a moderate CP–genome binding energy of ~7*k*_*B*_ *T*, which is unexpectedly low, because the en masse assembly mechanism is associated with strong CP–genome interactions. Moreover, the same analysis was carried out when lowering the solution pH to 5.2. In this case, the process was found to take place over a much longer time frame (~3,000 s), with *τ*_bind_ ≈ *τ*_struc_. Interestingly, this suggests that the system proceeds through a synchronous mechanism, where the evolution of the particle toward icosahedral symmetry and the addition of new CP dimers to the growing capsid occur simultaneously; in agreement with coarse‐grained simulation (Perlmutter et al., 2014). Furthermore, repeating pH 5.2 experiments at different temperatures, a binding activation energy of 20*k*_*B*_ *T* was obtained, indicative of a reaction‐limited process. Figure 2b shows a schematic representation of the free energy landscape of the CCMV assembly around ssRNA. As can be appreciated from the figure, the assembly reaction can be characterized in terms of CP–CP and CP–genome contacts. In this particular case of an en masse and synchronous pathway, Figure 2b visualizes how the driving force is first due to CP–genome interactions, and second led by CP–CP interactions.

### 2.3 Genome specificity scenarios

Genome specificity is one of the most intriguing features of viruses. It is revealed in very diverse ways for different viruses, sometimes exhibiting the finest and most precise mechanisms of nature. As we have already discussed, the electrostatic interactions formed between genome and CPs, are a major driving force in the viral assembly. However, these electrostatic interactions were early proven to be rather nonspecific, as demonstrated by in vitro assembly experiments using heterologous nucleic acids and even negatively charged polymers to generate VLPs (Bancroft, Hiebert, & Bracker, 1969; Hohn, 1969). Therefore, considerations of electrostatics alone do not provide an explanation for the remarkable characteristic of viruses to recognize their own genome; instead, it raises more questions.

#### 2.3.1 Packaging signals

The well‐studied plant virus, TMV, forms ~18 nm diameter rod‐like helical particles of ~300 nm in length composed of two components: the capsid protein (CP) and a single molecule of its ssRNA genome. TMV exhibits a very robust mechanism to control the packaging process. The TMV genome encodes a packaging or nucleation signal, which provides the desired specificity for CP capsomers to be recognized as the initiating point for assembly. TMV packaging signal (PS) is a short sequence that forms a loop of a well‐defined hairpin like‐structure (Zimmern, 1977; Zimmern & Butler, 1977). Somehow, CP capsomers (CP disks) are able to discriminate this region from many similar stem‐loops present in the TMV genome, which in principle is solely due to the higher affinity of the TMV CP for this PS. The robustness of this PS strategy is demonstrated when in vitro assembly is performed on modified genomes including two to four PSs (Eber, Eiben, Jeske, & Wege, 2015). In this scenario, wild‐type TMV particles can be distorted and form non‐linear particles. In particular, “tetrapods” are formed when four PSs are included in the ssRNA sequence. The TMV PS is so strong and specific that it can also be inserted into heterologous RNA leading to efficient packaging of any RNA into wild‐type TMV capsids. These results highlight how PS can regulate the initiation of assembly and selectivity in virology. Similarly, human immunodeficiency virus 1 (HIV‐1) selectively packages its genomic RNA during virus assembly. Although the HIV‐1 recognition mechanism is not fully understood (Comas‐Garcia, Davis, & Rein, 2016), recent studies suggest that the interaction of Gag (viral structural protein) with the PS of HIV‐1 has a nonelectrostatic component, which confers the desired specificity (Comas‐Garcia et al., 2017; Comas‐Garcia et al., 2018).

TMV and HIV‐1 assembly exemplify the robustness of encoding a well‐defined PS to trigger specific and selective capsid formation, while the rest of the genome does not seem to play a sequence‐specific role during the assembly process. However, viruses do not always show a clear single PS on their genome driving the assembly; this is particularly valid for spherical ssRNA viruses. Instead, a new strategy has been proposed to explain genome specificity of these viruses, highlighting the active role of the whole viral genome during packaging. It relies on the fact that multiple and dispersed specific interactions between the genome and the CPs take place during capsid assembly. Thus, multiple, disperse, PSs are represented by multiple secondary structure elements with CP recognition features. Based on these premises, a model of PS‐mediated assembly was applied using Gillespie algorithm simulations, to characterize the kinetics and assembly efficiency of this group of spherical viruses (Dykeman, Stockley, & Twarock, 2013a, 2014). The model relies on simple rules: CP capsomers interact with disperse PSs on the genome at different rates depending on CP–PS affinity; and the CP–CP capsomer interaction rates are determined by the free energy of CP–CP bonds. In addition, protein concentration must increase during assembly, known as protein ramp, as has been reported for bacteriophage Qβ assembly in vivo (Eigen, 2000). This novel theoretical framework solves the protein folding equivalent of Levinthal's Paradox for virus self‐assembly and explains the genome specificity and cooperativity of the process (Dykeman et al., 2014). The extent of the model has been recently reviewed by the authors (R. Twarock, Bingham, Dykeman, & Stockley, 2018; R. Twarock & Stockley, 2019). Furthermore, similar results have been found by coarse‐grained particle‐based simulations (Perlmutter & Hagan, 2015b). The authors identify more compact intermediates of assembly when adding PSs to the system; finding that a combination of one high affinity PS and several low affinity PSs leads to the highest assembly yields.

**Figure 3:**
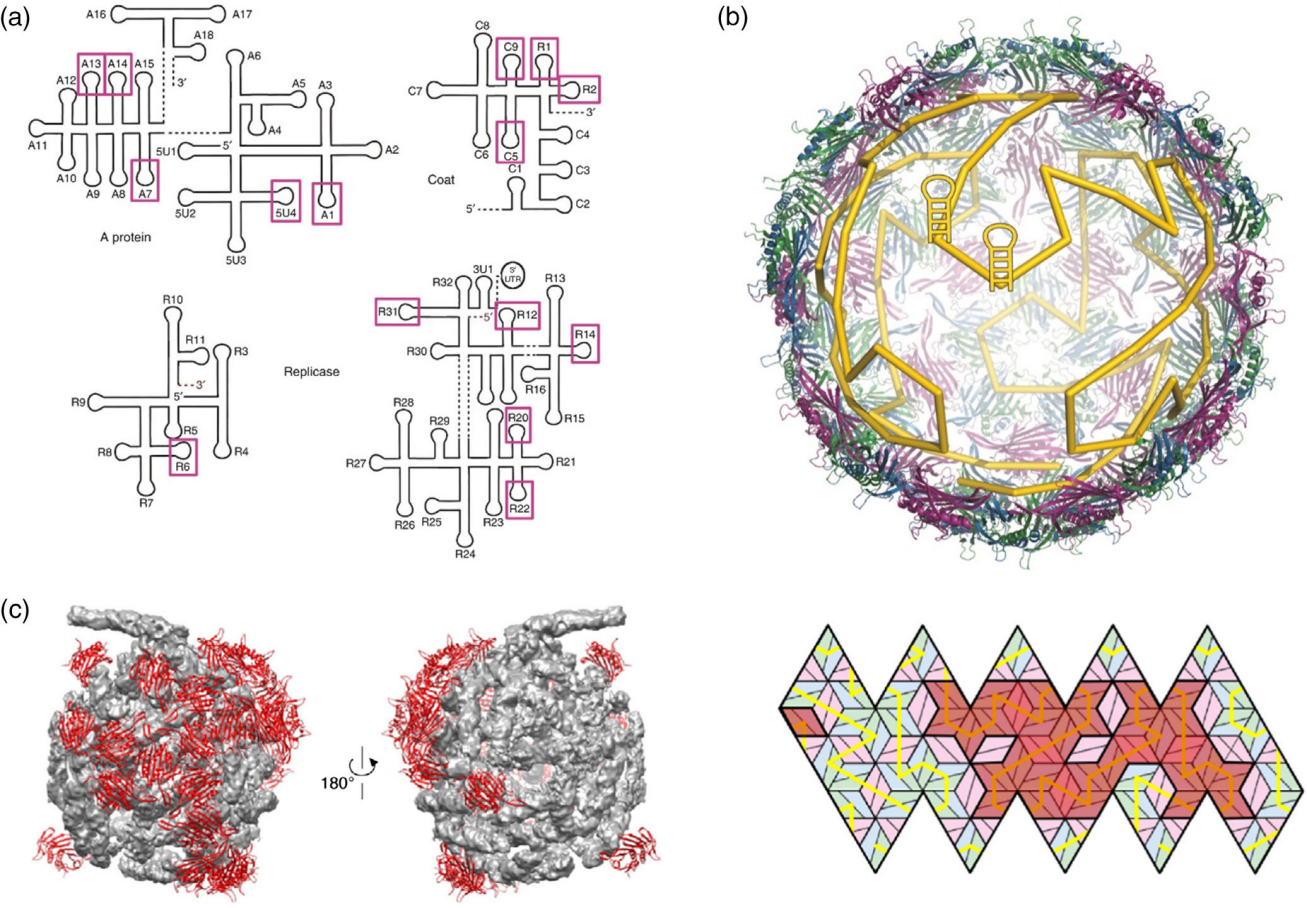
MS2 assembly predicted by the PS‐mediated model. (a) Representation of the MS2 genome with the 15 stem‐loops (magenta boxes) found in the asymmetric cryo‐EM reconstruction (Dai et al., 2017), and previously predicted to be PSs via HPA (Dykeman, Stockley, & Twarock, 2013). (b) Hamiltonian path representation of MS2 genome arrangement connecting binding sites inside the MS2 capsid. (a and b reprinted with permission from Twarock, Leonov, and Stockley (2018)). (c) Left, identified PSs by cryo‐EM reconstruction of MS2 particles at 8.7 Å resolution (Koning et al., 2016) are predominantly located in one half of the capsid; in agreement with predictions (right; Dykeman et al., 2013), showing that the positions of PSs bound to CPs (red rhombs) are also mainly located in one half of the capsid inner surface. (c reprinted with permission from Twarock, Bingham, et al. (2018)). CP, capsid protein; HPA, Hamiltonian path analysis; PS, packaging signal

The suitability of the PS‐mediated assembly model has been fully tested on the well‐characterized bacteriophage MS2, a positive‐sense ssRNA virus with a sphere‐like capsid of icosahedral symmetry. The MS2 genome was scrutinized using SELEX (systematic evolution of ligands by exponential enrichment), in order to identify protein‐binding sites on RNA (Figure 3a), combined with Hamiltonian path analysis (HPA) to predict aspects of genome organization (Figure 3b; Dykeman, Stockley, & Twarock, 2013b). Disperse PSs were also identified on the MS2 genome using crosslinking immunoprecipitation and desorption/ionization mass spectrometry (Rolfsson et al., 2016), showing excellent agreement with PSs positions proposed in Dykeman et al. (2013b). In parallel, the asymmetric cryo‐EM reconstruction of MS2 particles at 8.7 Å resolution (Koning et al., 2016), and the subsequent reconstruction at 3.6 Å (Dai et al., 2017) revealed the structures of both the protein shell and the asymmetric genome arrangement. The 15 high affinity PSs predicted by Dykeman et al. (2013b), and therefore expected to be conserved in every viral particle, are found in contact with CP capsomers in both MS2 cryo‐EM reconstructions. In addition, the asymmetrical arrangement of the MS2 genome derived from the HPA is also seen in the mentioned EM structures (Figure 3c; R. Twarock, Leonov, & Stockley, 2018). These remarkable results corroborate the suitability of the PS‐mediated assembly model combined with HPA to characterize MS2 assembly. Furthermore, disperse PSs have been recently identified in other viruses, such as satellite tobacco necrosis virus (Patel et al., 2015; Patel et al., 2017), human Parechovirus (Shakeel et al., 2017), hepatitis C virus (Stewart et al., 2016), and HBV (Patel et al., 2017). Thus, even though PSs may play distinct roles in different viruses during the assembly process, all of them could share the same basic mechanism: disperse sites in their (pre)genomes with affinity for their associated CPs drive the efficient formation of capsids with the optimal geometry.

#### 2.3.2 Overall RNA topology effects

Packaging signals are not the only features to take into account when studying genome specificity during viral self‐assembly. Concomitant with PS‐mediated studies, efforts have been devoted to decipher the role played by nonspecific electrostatic interactions between CPs and viral genomes, and by physical properties of RNA genomes such as length, degree of branching, and stiffness. Such studies are motivated by experimental observations for certain viruses that point toward a marginal or nonexisting role of sequence specificity. For instance, in vitro assembly experiments have shown that CP of HBV has no clear preference packaging genomic RNA over heterologous RNA of equal length (Porterfield et al., 2010). Similarly, competition assays in which a limited amount of CCMV proteins is mixed with an equal amount of CCMV and Brome mosaic virus (BMV) genomes, showed that CCMV particles are preferentially assembled around BMV RNA, that is, BMV RNA outcompetes CCMV cognate RNA for CCMV CPs (Comas‐Garcia, Cadena‐Nava, Rao, Knobler, & Gelbart, 2012). This example highlights the impact of RNA topology, as BMV RNA has a more compact arrangement than the cognate RNA of CCMV (Erdemci‐Tandogan, Wagner, Schoot, Podgornik, & Zandi, 2014). In particular, it was proposed that an increasing degree of RNA branching produces a gain in assembly efficiency. Specifically, the RNA with larger number of branching junctions, and therefore with a more compact arrangement, exhibits an advantage under competitive packaging conditions.

**Figure 4:**
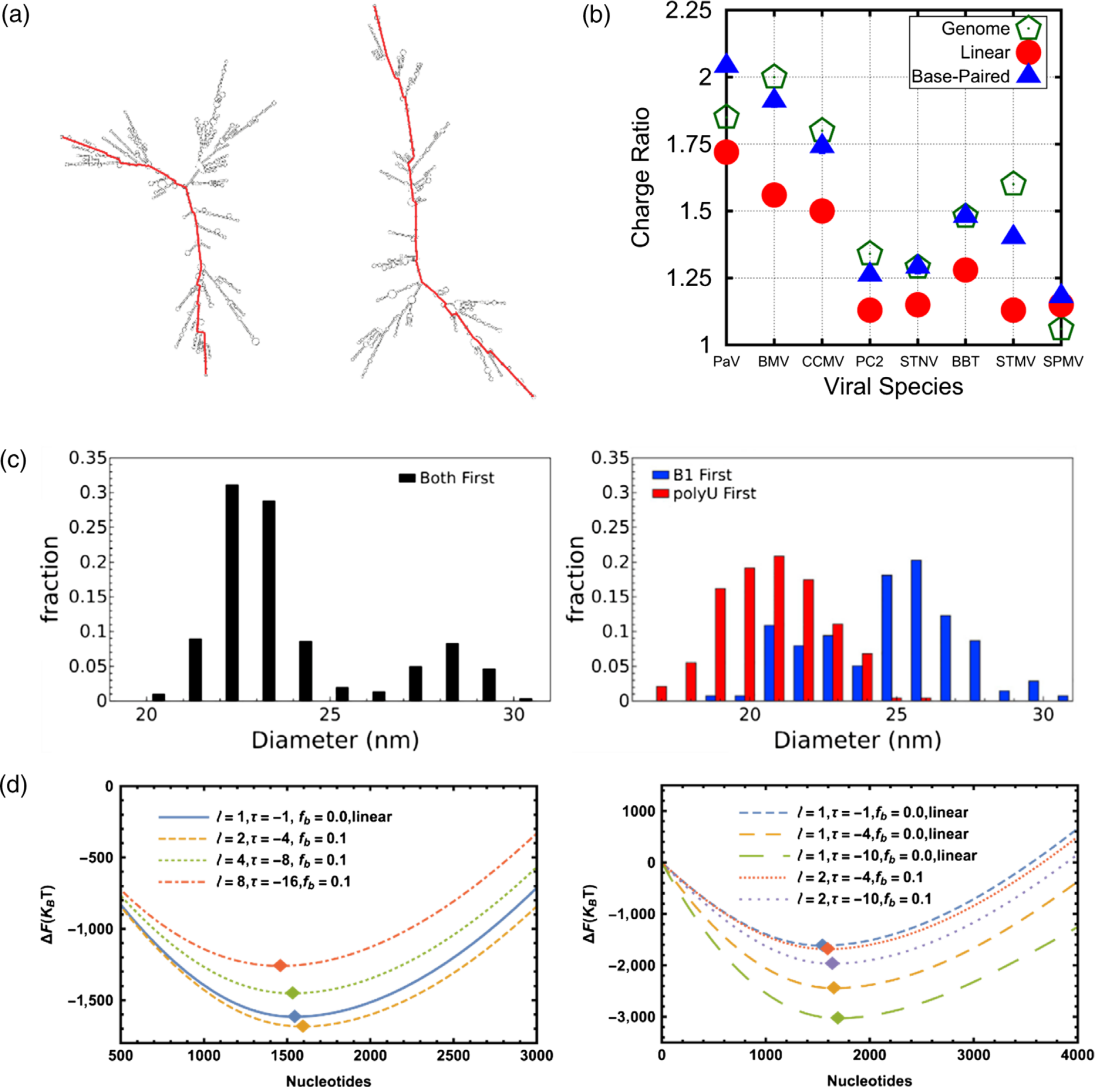
Contributions of RNA topology to viral assembly. (a) Comparison of the secondary structure of BMV RNA (left structure), and a nonviral random RNA sequence (right structure) with equal numbers of bases and base proportions. The maximum ladder distance, that is, the number of basepairs crossed along the trajectory between the two most distant hairpin loops, of both structures are 207 and 354, respectively, represented as red lines. (Reprinted with permission from Ben‐Shaul and Gelbart (2015)). (b) Plot of the charge ratios (genome charge/CP charge) calculated for several viruses (green pentagons), and predicted for linear polyelectrolytes (red circles) and model nucleic acids with 50% base‐pairing (blue triangles). (Reprinted with permission from Perlmutter et al. (2013)). (c) Size distributions of the VLPs formed from competitive self‐assembly. Left, competition assay in which polyU (~22 nm peak) and BMV RNA (28 nm peak) are mixed simultaneously. Right, competition assays altering the order of addition of polyU and B1 (BMV RNA). (Reprinted with permission from Beren, Dreesens, Liu, Knobler, and Gelbart (2017)). (d) Free energy of encapsidation for linear and branched polynucleotides as a function of chain size. Left, effect of stiffness and charge density. Right, a closer look at the changes in charge density. Parameters are *l* (Kuhn length), *τ* (charge within one Kuhn segment), and fb (fugacity). (Reprinted with permission from Li, Erdemci‐Tandogan, van der Schoot, and Zandi (2018)). BMV, Brome mosaic virus; CP, capsid protein; VLP, virus‐like particle

The observation that more compact RNA structures could lead to more efficient packaging is supported by simulation studies (Perlmutter, Qiao, & Hagan, 2013; Singaram, Garmann, Knobler, Gelbart, & Ben‐Shaul, 2015) and mean‐field theory calculations (Erdemci‐Tandogan et al., 2014; Erdemci‐Tandogan, Wagner, van der Schoot, Podgornik, & Zandi, 2016; Li, Erdemci‐Tandogan, Wagner, van der Schoot, & Zandi, 2017). These studies show that the amount of RNA bases that can be packed by a given protein shell depends on how efficient RNA secondary structures are formed. In fact, it has been demonstrated that nonviral RNA is in general less compact than the viral RNA genomes (Figure 4a), when comparing RNA molecules with the same length and similar amount of base‐paring (Ben‐Shaul & Gelbart, 2015; Bruinsma, Comas‐Garcia, Garmann, & Grosberg, 2016; Gopal, Zhou, Knobler, & Gelbart, 2012; Yoffe et al., 2008). Perlmutter and coworkers also performed coarse‐grained particle‐based simulations for several specific viruses, considering genome secondary structure and total charge of CPs (Perlmutter et al., 2013). Figure 4b shows how these predictions compare to values of charge ratio (genome charge/CP charge) when genome base‐paring is included. The concepts of nonspecific electrostatic interactions and RNA branching provide further insights into our understanding of genome packaging by considering intramolecular charge repulsion compensation, between CPs and ssRNA, and by accounting for the compact conformations of viral genomes. Therefore, these findings indicate that sequence‐specific protein‐RNA interactions are not the only mechanism that leads to genome specificity. In the next section, we will discuss how RNA topology also influences the stability and mechanical properties of the formed viral particles, as a complementary approach to understand viral packaging.

In order to further investigate the suitability of the above presented theories, Beren and coworkers recently performed in vitro assembly experiments using CCMV CPs and polyU, an ssRNA molecule only composed of uridylic acid that lacks secondary structure (Beren et al., 2017). Following the same strategy reported by (Comas‐Garcia et al., 2012), head‐to‐head competition experiments were carried out to test the preference of CCMV CP for its cognate RNA over the less compact and unfolded polyU molecules. Surprisingly, competition assays showed that polyU outcompetes viral RNA for CP. Even when using BMV RNA, which outcompetes CCMV cognate RNA, polyU wins the assembly competition for CCMV CPs. These intriguing results also lead to *T* = 2‐sized particles (~22 nm) when polyU is packed by CCMV CPs, in contrast with the well‐characterized *T* = 3 of CCMV wild‐type particles (28 nm; Figure 4c, left graph). Somehow, polyU molecules of ~3,000 nucleotides, which is a similar length to CCMV RNA and BMV RNA, form smaller VLPs than wild‐type particles, even though their 3D size (hydrodynamic radius) is larger, in disagreement with the RNA topology theories presented above. In addition, the authors also find that the order in which RNA molecules are added to the assembly reaction critically determines the outcome of the experiments (Beren et al., 2017), as opposed to the observations made by (Comas‐Garcia et al., 2012) for other RNA molecules. Figure 4c shows the size distribution of VLPs obtained from the competitive self‐assembly reactions when BMV RNA (B1) and polyU are added simultaneously (left graph), and when the order of mixing was altered (right graph; Beren et al., 2017). These experiments show for the first time, how a linear polymer (polyU) outcompetes branched ones (viral RNA), while keeping all other chain quantities equal, and are not what would be predicted by the previously mentioned experimental work, simulations and theoretical calculations.

These observations emphasize that the role of RNA topology in the self‐assembly of spherical ssRNA viruses is still not fully understood. In this context, Van der Schoot and Zandi already suggested the important role of balancing Kuhn length and linear charge density distribution in order to predict the free energy gain of polymer encapsulation (van der Schoot & Zandi, 2013). The Kuhn length of ssRNA at neutral pH in the presence of monovalent salt can vary between 1 and 2 nm (H. Chen et al., 2012), while that of dsRNA is bigger than 120 nm (Abels, Moreno‐Herrero, van der Heijden, Dekker, & Dekker, 2005; Kebbekus, Draper, & Hagerman, 1995), that is, dsRNA is much stiffer than ssRNA. Moreover, a further difference between single‐stranded and double‐stranded RNA molecules is the linear charge density, which is double for dsRNA, due to base‐paring. Accounting for these physical properties of RNA, Li and coworkers recently performed new calculations applying mean‐field theory (Li, Erdemci‐Tandogan, et al., 2018). The authors investigated the impact of RNA stiffness, by exploring changes in the mean Kuhn Length of the chains, and the effect of base‐paring on the distribution of the linear charge density. Interestingly, the effect of stiffness overshadows the charge density contribution, when looking at free energy gains upon RNA encapsidation as a function of the polynucleotide size (Figure 4d). It also highlights the availability, within the free energy landscape, of certain conditions where the packaging of linear RNA becomes more favorable than for branched RNA (Figure 4d).

Thus, RNA base‐pairing seems to have competing effects: (a) it makes RNA stiffer, increasing the work of packaging that must be overcome by the CPs; however, (b) it introduces branching junctions, hence, compactness, and (c) it enhances the charge density, both promoting efficient assembly. These results provide new insights into how linear ssRNA molecules can outcompete branched ones under assembly competition conditions. In addition, it is important to mention that these calculations are in full agreement with the already mentioned studies (Erdemci‐Tandogan et al., 2014, 2016; Li et al., 2017; Perlmutter et al., 2013; Singaram et al., 2015), where the implications of RNA stiffness and charge density were not explored. Despite these new insights the models still predict that branched polymers have a competitive edge over linear ones, even when the effects of changes in stiffness and charge density are considered (Li, Erdemci‐Tandogan, et al., 2018). This reveals that the existing theoretical frameworks do not yet capture essential aspects of assembly and clearly there is a need for both more (and maybe different) experiments as well as further developments in modeling/theory in order to understand how viral self‐assembly occurs.

## 3 MECHANICAL STABILITY OF VIRUSES

The material properties and mechanical stability of viruses are governed by several factors, such as for instance, (a) the inter‐ and intramolecular interactions (covalent, noncovalent, electrostatic, hydrophobic, etc.) of the capsid proteins (CPs; Ashcroft et al., 2005; Mateo, Diaz, Baranowski, & Mateu, 2003; Mateu, 2009, 2012, 2013; Reguera, Carreira, Riolobos, Almendral, & Mateu, 2004; Roos et al., 2012); (b) the protein(CP)‐nucleic acid interactions (mostly electrostatic interactions at the capsid interior‐nucleic acid interface; Devkota et al., 2009; Ni et al., 2012; Rao, 2006; Reade, Kakani, & Rochon, 2010; Schneemann, 2006; Snijder et al., 2013); (c) prestress resulting from capsid architecture or pressure associated with genome encapsidation (Baclayon et al., 2011; Carrasco et al., 2011; Klug, Roos, & Wuite, 2012; M. Marchetti, Wuite, & Roos, 2016); (d) stabilizing molecular interactions during assembly or maturation; for example, interaction with metal ions, scaffolding proteins, or enzymatic reactions, and so on (Li, Roy, Travesset, & Zandi, 2018; Perera & Kuhn, 2008; Persson, Tars, & Liljas, 2008; Plevka et al., 2009; P. E. Prevelige & Fane, 2012; Saugar et al., 2010); (e) entropic stabilization through capsid breathing and self‐healing (C. Chen, Wang, & Zlotnick, 2011; de Pablo, Hernando‐Perez, Carrasco, & Carrascosa, 2018; J. Lin et al., 2012; Reisdorph et al., 2003; Valbuena & Mateu, 2015). There is certainly overlap between these factors, and typically viruses are stabilized and sometimes also destabilized by a combination of them. This variety in properties influencing capsid stability makes its study a diverse field. The insights gained are not only useful for preventing or reducing viral infectivity, but also for the generation of artificial, hollow supramolecular assemblies (nanocages) for various uses in bio‐nanotechnology, pharmacology, and materials sciences.

### 3.1 AFM‐based nanoindentation experiments

**Figure 5:**
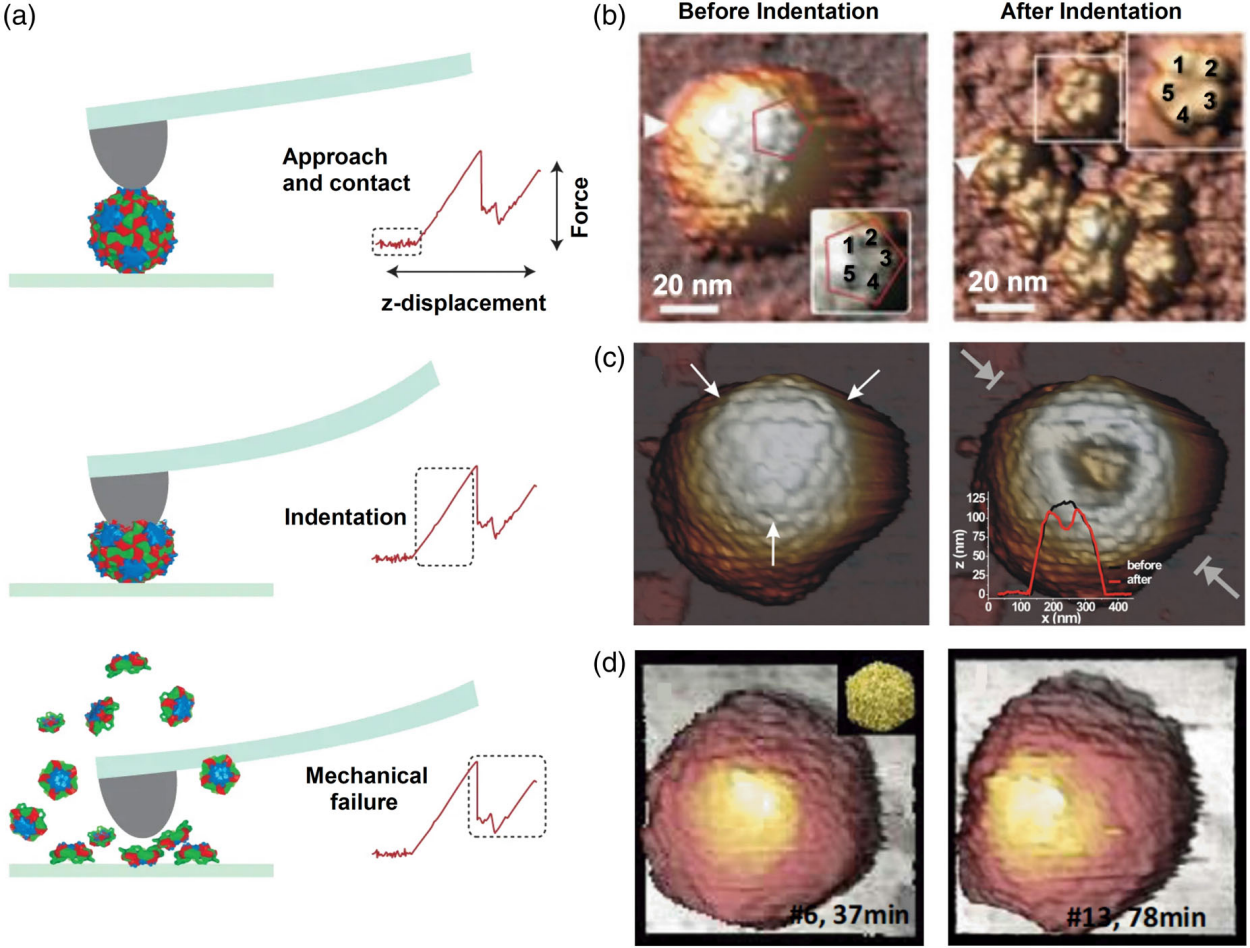
Response of virus particles to indentation forces. (a) Scheme of AFM nanoindentation experiment. (b–d) Examples of particles showing different effects upon indentation. (b) Mechanical failure of picorna‐like Triatoma virus (TrV). (Panels adjusted from Snijder et al. (2013); with permission from the publisher). (c) Irreversible deformation of herpes simplex virus Type 1 (HSV1). (Panels adjusted from Klug et al. (2012); with permission from the publisher). (d) Reversible deformation of T7 bacteriophage. The particle shows plastic deformation immediate after the indentation, but resumed its structure after ∼36 min (right panel). (Panels adapted from de Pablo et al. (2018); with permission from the publisher). AFM, atomic force microscopy

In order to characterize the mechanical properties of viruses and to capture particle‐to‐particle variations in these properties AFM‐based force spectroscopy experiments have been developed (Ivanovska et al., 2004; Roos et al., 2010). A detailed experimental protocol of such AFM based nanoindentation experiments can be found in (Guo & Roos, 2019). Briefly, the viruses are first attached to a surface. It is important to have an attachment which is not so strong that the particle will be deformed, but it must not be too weak either so that it can roll over during the imaging/indentation. Next, the particle is localized by AFM imaging and a force distance curve (F‐D) is taken on the center of the particle (Figure 5a). Another image of the particle is taken after the indentation to reveal the state of the particle after deformation. Typically, three different scenarios can be distinguished upon indentation. Depending on the viral mechanical architecture, the particle can exhibit a total collapse (Snijder, Uetrecht, et al., 2013; Figure 5b), or irreversible structural deformation whereby (a part of) the shell stays intact (Klug et al., 2012; Figure 5c), or self‐recovery to its original size (de Pablo et al., 2018; Figure 5d). Upon analyzing the F‐D curve one can deduce the stiffness of the particle (from the slope of the indentation curve), the ultimate strength of the particle (from the breaking force, if applicable), and the indentation depth (a mark of the deformability). In addition, using elasticity models one can also obtain an estimate for the intrinsic material properties in terms of Young's Modulus (Roos et al., 2010).

### 3.2 Mechanical properties linked to viral assembly and disassembly

Initially, started as a curiosity driven quest (Ivanovska et al., 2004), nanoindentation‐based mechanical studies of viruses is now a wider applied physical virology technique. The determination of mechanical properties of viruses is not only providing information about capsid stability, but also unveiling physicochemical and biological links between different stages in a virus‐life cycle (Carrasco et al., 2006; Kol et al., 2007; Roos et al., 2010; Roos et al., 2012; Snijder, Uetrecht, et al., 2013). For instance, Carrasco et al. (2011) have investigated the empty φ29 bacteriophage proheads using AFM‐based nanoindentation experiments in combination with coarse‐grained simulations. In this work, a twofold anisotropic stiffening of the capsid along the short axis was observed. The authors concluded that during assembly curvature of the capsid protein is induced by the scaffolding proteins resulting in a structural stress within the proheads. This stress can then later be used as a trigger for DNA release through the tail region. Around the same time, Baclayon et al. (2011) have investigated Norwalk virus‐like particles (NVLPs) to scrutinize the role of the protruding domain of the NVLP capsid protein in the particle's stability. It turned out that the presence of this domain generated a stabilizing prestress in the shell. This finding shows that the protruding domains are not only responsible for specific cell binding during the infection cycle, but also have a role in survival of the particle when exposed to stress factors.

Next to structural components in the capsid, also the internalized genome can influence disassembly. Nanoindentation of the picorna‐like Triatoma virus was performed in combination with Mass Spectroscopy to scrutinize its stability (Snijder, Uetrecht, et al., 2013). Under the indentation force, the particle exhibited mechanical failure (Figure 5b). After particle disruption, AFM imaging revealed that the particle disassembled into its single penton structural units. While doing a pH sweep, it turned out that at neutral pH, the genome stabilizes the particle, but that at alkaline pH, it is destabilizing the capsid. This behavior seems to be linked to the infectious pathway in which the particle passes the alkaline parts of the gut before infection occurs. Genome induced stress also seems to play a role in human adeno virus (HAdV) disassembly (Ortega‐Esteban et al., 2013; Ortega‐Esteban et al., 2015). By continuous imaging of the particle with the AFM tip, at a certain moment fatigue occurred and the pentons started coming of the infectious particles. As these prolonged stresses are not likely to be the sole factor in disassembly during infection, other factors must facilitate this process. It turned out that binding of HAdV to the host cell receptor integrin ανβ5 plays a pivotal role in the first stage of infection and particle destabilization (Snijder et al., 2013). By binding to integrin, a conformational change occurs in the penton base, thereby loosening it and facilitating the later release of the penton base. Minor capsid proteins, such as pUL17 and pUL25 of herpes simplex virus Type 1, can also influence particle stability (Snijder et al., 2017). Nanoindentation experiments with wild‐type and deletion mutant particles revealed that these minor capsid proteins, which bind specifically close to the fivefold axis, bring stability to these weakest parts of the capsid. Finally, complex capsid structures such as the multilayered rotavirus turn out to have different mechanical properties for each layer, fitting with the needs for protection (stiff outer layer) and genome expression (flexible middle layer) at different stages of the life cycle (Jimenez‐Zaragoza et al., 2018).

### 3.3 Mechanical properties linked to genome encapsidation

**Figure 6:**
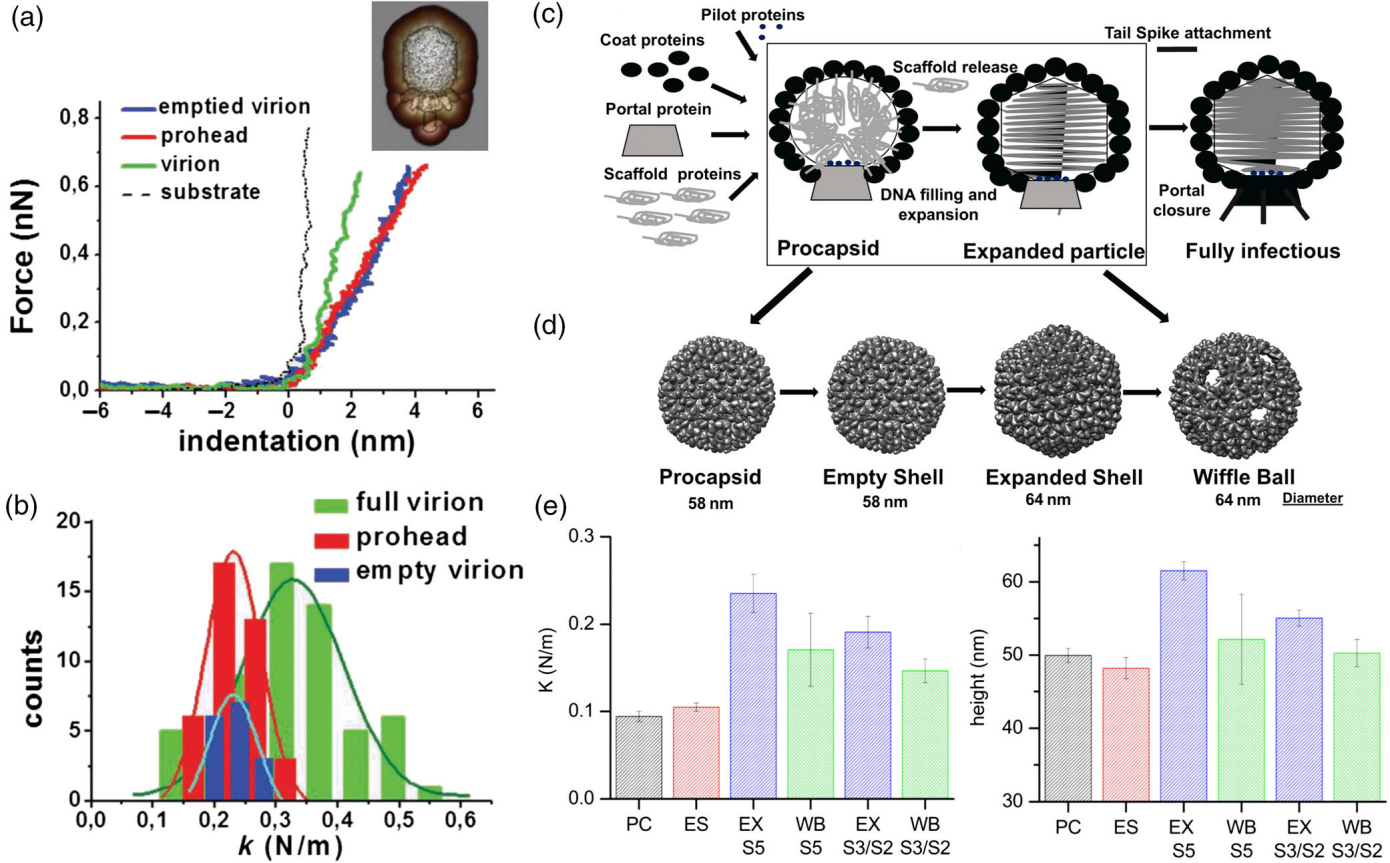
Effect of genome encapsulation and maturation. (a) Force–indentation curves obtained from an empty particle, a prohead and a complete virion of the φ29 bacteriophage. Inset is a typical AFM image of a φ29 bacteriophage virion, with a superimposed reconstruction from EM. (b) Calculated spring constant from the experiments in (a). (Panel a and b taken from Hernando‐Perez et al. (2012), with permission from the publisher). (c) Scheme of in vivo maturation of bacteriophage P22. (d) P22 VLP reconstructions at different stages. (e) The measured spring constant (left) and height (right) of capsids in different stages. PC: procapsid, ES: empty capsid, EX: expanded shell for five‐, three‐, and twofold symmetry (S5, S3, and S2, respectively). (Panels taken from Kant et al. (2018); with permission from the publishers). AFM, atomic force microscopy

As mentioned before, the encapsulation of genetic material can have a significant impact on capsid morphology and stability. Therefore, capturing the mechanical effects of the presence of the genome in viral shells is a way to gain insight into the physicochemical aspects of virus survival and infectivity. Nanoindentation experiments on MVM revealed anisotropic mechanical properties along the different symmetry axes in the DNA containing particles (Carrasco et al., 2006). Comparing with the response of empty capsids, it was concluded that the anisotropic reinforcement was mediated by DNA packaging. This interpretation was also supported by the crystal structure, in which short DNA patches at the inner capsid wall were visible. Combining with finite element modeling, the authors concluded that the MVM particle is not reinforced at their fivefold symmetric axis, in order to allow for DNA release during infection. In a follow‐up study (Carrasco, Castellanos, de Pablo, & Mateu, 2008), specific mutation in the capsid protein were introduced to remove the DNA–capsid interaction. The measured change in material properties of the mutated particles, corroborated their earlier interpretations on the DNA mediated anisotropic reinforcement of the shell. As mentioned above, Triatoma virus shows an intricate interaction between capsid and genome with stabilizing interactions at neutral pH but destabilizing ones at higher pH (Snijder, Uetrecht, et al., 2013). This seems also directly related to disassembly. A change in stiffness upon genome encapsidation was furthermore observed for bacteriophage φ29 (Hernando‐Perez et al., 2012; Figure 6a,b). While the scaffolding protein did not play a significant role in the mechanical stability of the capsid, the presence of DNA resulted in a genome induced pressure of 40 ± 20 atm inside the virion. For SV40, genome encapsidation did not affect the stiffness, but it increased the particle resilience against large deformations (van Rosmalen, Li, Zlotnick, Wuite, & Roos, 2018). Furthermore, the VLP material properties were affected by the addition of the reducing agent DTT and calcium‐ion chelating EDTA, with, respectively, a reduced resistance to mechanical stress and a softening of the shell as result. In a recent study on adenovirus stability (van Rosmalen, Nemerow, Wuite, & Roos, 2018), particles with a mutation in precursor protein VI were studied. It was revealed that the mutant exhibits a factor of two increase in stiffness, while the infectivity remains constant. The stiffening seems to be the result of the presence of pVII in the mature, mutant capsids, leading to DNA crosslinking. Interestingly, a study on HIV particles showed that sometimes not the genome itself (with or without crosslinking proteins), but reverse transcription of the genome is generating a pressure that leads to disassembly of the viral particle (Rankovic, Varadarajan, Ramalho, Aiken, & Rousso, 2017). Overall, it seems that there is a correlation between the Young's modulus and the manner of encapsidation of viruses. While viruses that self‐assemble around their genome possess a relatively low Young's modulus, the modulus is higher for particles that self‐assemble empty and that use a packaging motor to encapsidate the genome (Roos et al., 2010). The latter method is expected to lead to higher internal pressures and therefor the necessity for stronger shells to hold the pressurized genome.

### 3.4 Mechanical properties linked to viral maturation

**Figure 7:**
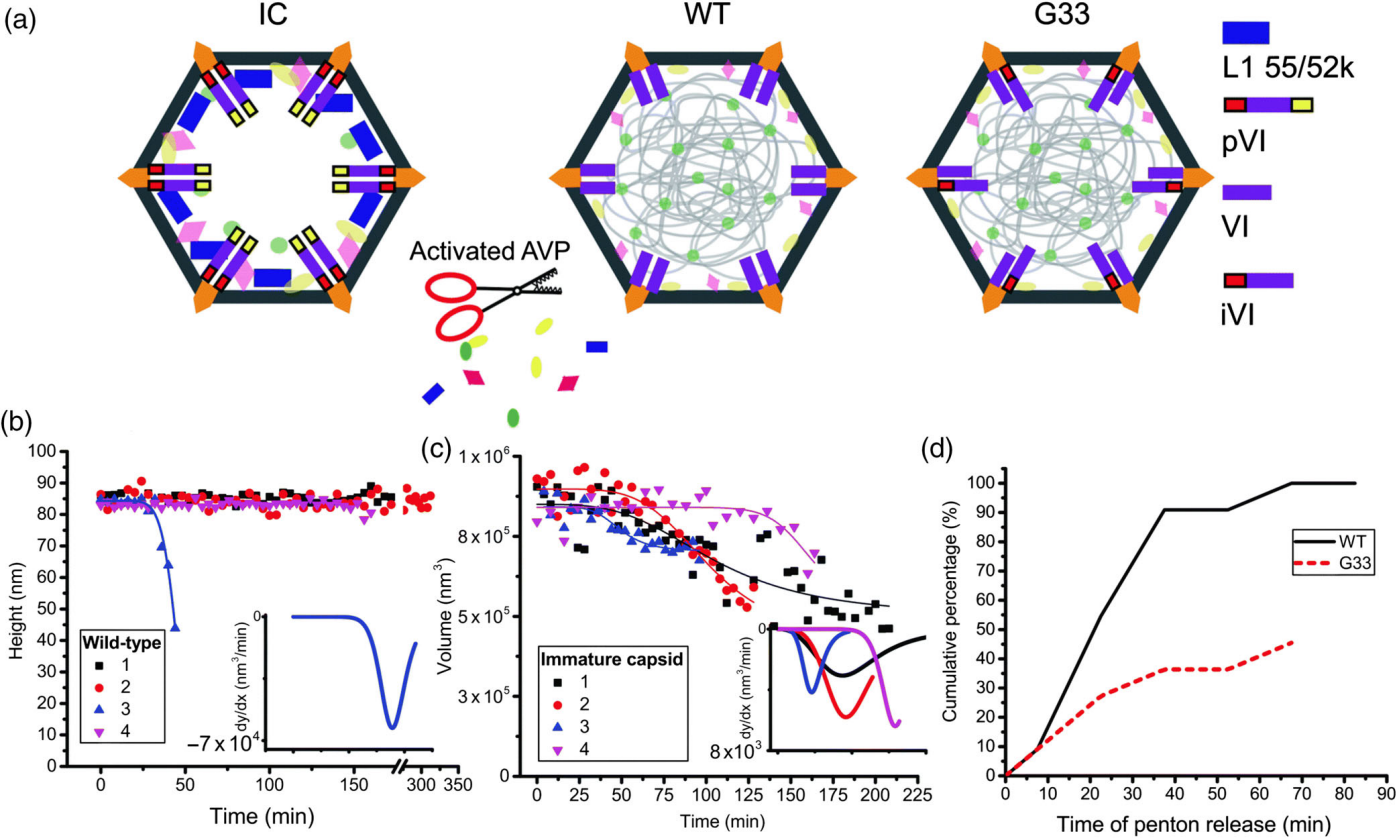
Mechanical fatigue of human adenovirus immature (IC), mature Ad5 (WT), and G33 mutant (G33) capsids. (a) Schematic representation of IC, WT, and G33 particles with relevant core component composition. (b) Plot of change in height over time for constant AFM imaging of WT particles. Inset: first derivative of sigmoidal fit from the plot in (b). (c) Plot of change in volume over imaging time for IC particles. Inset: first derivative of sigmoidal fit of curves in main panel. (d) Comparative representation of cumulative percentage of penton release of WT and G33 particles for AFM tip induced fatigue experiments. (Reproduced from Denning et al. (2019) with permission from The Royal Society of Chemistry). AFM, atomic force microscopy

Capsids of viruses that undergo maturation typically go through a set of conformational changes. By studying the mechanical changes accompanying this process, insights are provided into these maturation steps. To scrutinize these steps in bacteriophage HK97, which is a *λ*‐like phage, the particle was studied by AFM‐based imaging and nanoindentation (Roos et al., 2012). It turned out that maturation results in an increase in mechanical stability of the capsid in three different ways: increasing Young's modulus of the mature capsid, increasing capsid strength, and increasing resistance to material fatigue. Phage *λ* undergoes maturation induced changes at the same positions in the capsid as phage HK97, but the changes in the latter are crosslinking of the capsid proteins and in the former an extra protein is added at those positions. It was shown that phage *λ* also increases its strength during maturation (Hernando‐Perez, Lambert, Nakatani‐Webster, Catalano, & de Pablo, 2014). So while these related phages have found completely unrelated ways of reinforcing their capsid during maturation, they both reinforce the same places in the capsid, indicating that these locations are the weak spots in the capsid. A comparative study of adenoviral maturation revealed that the genomic core of an immature particle shows a stiffer response than the mature core (Ortega‐Esteban et al., 2015). The decondensation of the core upon maturation makes it more flexible and this flexibility is thought to facilitate genome release. This is, however, not the only factor in genome release, as first the pentons need to be removed from the capsid. The proteolytic cleavage of preprotein VI during maturation is essential for this penton destabilization, as revealed by imaging based fatigue experiments (Denning et al., 2019; Figure 7).

While bacteriophage HK97 and *λ* undergo a stabilizing transition during maturation, it is the other way around for HIV. Immature HIV particles are more than 14‐fold stiffer than mature particles, and this large difference is primarily mediated by the HIV envelope cytoplasmic tail domain (Kol et al., 2007). The authors showed that this stiffness switch is essential to inhibit immature particles to enter host cells. Bacteriophage P22 is known to undergo a series of intermediate stages during maturation (Parker & Prevelige Jr., 1998). While mimicking these intermediate stages, by application of thermal and chemical stresses, it was shown that the rigidity and brittleness increased after maturation induced expansion, as predicted by continuum elasticity theory (Kant et al., 2018; Figure 6c–e). For another bacteriophage, phage T7, it turned out that stiffness was not an ideal parameter to describe viral stability, but that the particle fragility provides a better characteristic (Hernando‐Perez et al., 2014). This shows that various material properties can be used in order to elucidate the mechanics and stability of viral particles.

## 4 CONCLUSION

Viruses possess extraordinary features and functions. They have evolved in close dependency to their living hosts, influencing the development of life. Therefore, the study of viral systems has impacts beyond virology. Viral systems present many *vital* characteristics found in living organisms. For instance, the capacity of viral components to self‐assemble into supramolecular structures, to recognize specific targets, and to possess a high adaptability to environmental conditions. In addition, viruses are formed from selective biomacromolecules that exhibit the functions and specificity needed to hijack and dominate cellular processes. Recently, studies on viruses and their derived VLPs have grown drastically, not only to better understand viral and nonviral systems, but also due to the opportunities these systems have opened up in areas such as nanomedicine and (bio)nanotechnology. Here we have discussed how viral self‐assembly is relying on fine‐tuned interactions between the capsid proteins and the viral genome. The balancing of these interactions, CP–CP and CP–genome, implicitly conditioned by the surrounding environment, will ultimately determine the assembly pathway(s). Certain viruses support specific packaging by including conserved genomic sequences that allow CPs to discriminate the cellular genetic material to ensure the formation of virions. Moreover, the PS‐mediated assembly theory gives a comprehensive understanding for the successful formation of optimal capsid geometries within the conformational assembly landscape. However, other viruses seem to assemble showing residual or absence of sequence specificity, at least under in vitro conditions, where genome topology has proven to be a potential candidate to understand the assembly of these kind of viruses. Viruses and their derived VLPs are found within a broad range of material properties. Mechanics is not only an essential factor in genome encapsidation and maturation, but also in self‐assembly and disassembly. In addition, viral systems have been proven to be highly dynamic, showing the capability to modulate stability during infection, which highlights their remarkable conformational plasticity and adaptability.

The discussed new insights in self‐assembly and mechanics show how valuable physical virology approaches are. The single‐particle methods and techniques presented here, such as AFM, EM, resistive‐pulse sensing, and CDMS; in combination with bulk methods, simulations, and theoretical calculations, have proven to be a good combination of approaches to shed light onto the molecular basis of viral systems. Still a lot of open questions remain, and there is an urgent need for innovative methods in order to finally elucidate the mechanisms behind assembly. In this respect, novel approaches such as magnetic tweezers, optical tweezers, acoustic force spectroscopy, and high‐speed AFM, which present advances in temporal and spatial resolution, seem promising techniques to follow viral assembly in real time, and at the single‐particle level. A very recent example of this is assembly studies by optical tweezers (K. D. Marchetti et al., 2019). With these new techniques and approaches, it is expected that in the coming years our understanding of viral self‐assembly and mechanics will be further deepened. The hereby newly generated insights will likely not only advance fundamental science, but also applications of viruses and VLPs.

## CONFLICT OF INTEREST

The authors have declared no conflicts of interest for this article.

## AUTHOR CONTRIBUTIONS

**Pedro Buzón**: Writing‐original draft and writing‐review and editing‐Equal. **Sourav Maity**: Writing‐original draft and writing‐review and editing. **Wouter Roos**: Conceptualization; supervision; writing‐original draft; and writing‐review and editing.

## RELATED WIREs ARTICLES

Synthetic virology: Engineering viruses for gene delivery

Viral chemistry: The chemical functionalization of viral architectures to create new technology

Synthetic plant virology for nanobiotechnology and nanomedicine

Physical, chemical, and synthetic virology: Reprogramming viruses as controllable nanodevices
